# Sex Differences in Medical Specialist Physicians’ Electronic Health Record In-Basket Workloads and the Implications for Compensation and Equity: Retrospective Cross-Sectional Study

**DOI:** 10.2196/79172

**Published:** 2026-02-18

**Authors:** Ashwini Nadkarni, Laura Glick, Molly Becker, Michael Healey, David Stein, Mahyar Heydarpour, Lisa S Rotenstein

**Affiliations:** 1 Brigham and Women's Hospital Boston, MA United States; 2 Massachusetts General Hospital Boston, MA United States; 3 Suffolk University Boston, MA United States; 4 University of California, San Francisco San Francisco, CA United States

**Keywords:** Electronic health records, In-basket messaging, Physician workload, Sex differences, Relative value units

## Abstract

In this retrospective cross-sectional study, we assessed sex differences in in-basket messages generated by outpatient workflows among internal medicine specialists as compared to relative value units; we found that female physicians had a greater burden of in-basket work for each unit of paid clinical care.

## Introduction

Female physicians in primary care receive higher volumes of patient and staff messages in the electronic health record (EHR) in-basket [1,2]. However, little is known on whether sex differences extend into medical specialties and impact on workload disparities relative to compensated care. In this cross-sectional study, we assessed sex differences in in-basket messages generated by outpatient workflows among internal medicine specialists as compared to relative value units (RVUs). We aimed to clarify whether female medical specialty physicians experience disproportionately greater uncompensated electronic work for each unit of paid clinical care.

## Methods

### Ethical Considerations

We followed the Strengthening the Reporting of Observational Studies in Epidemiology (STROBE) cross-sectional study guidelines. The Mass General Brigham Institutional Review Board deemed the study exempt (2021P001356). The informed consent requirement was waived because the study involved secondary analysis of deidentified EHR data and posed minimal risk to participants. Participants did not receive compensation. Although prior literature refers to “gender” differences, our analysis examined sex differences, so we used the terms “female” and “male.” We conducted the study at a large, academic medical center. All attending physicians actively practiced in outpatient specialty practices with measurable in-basket activity during the study period.

### Study Procedures

We collected monthly, ambulatory in-basket physician data and patient characteristics for each attending physician’s patient panel from Epic for January through March 2021 and normalized monthly in-basket burden measures by monthly RVUs. Patients are generally assigned to physicians based on availability and patient preference. We obtained physician sex, academic rank, and practice years from the public institutional directory, which reports sex as male or female; no physicians were listed as nonbinary. We categorized specialties as those including a substantial procedural component (cardiology, gastroenterology, and pulmonology) versus those predominantly not procedural (genetics, geriatrics, hematology, immunology, infectious disease, nephrology, palliative care, rheumatology, and sleep medicine).

### Statistical Analysis

We compared continuous variables using Wilcoxon rank sum tests, and categorical variables using chi-square tests or Fisher exact tests when expected cell counts were small. To assess sex differences in in-basket burden, we fit multivariable linear regression models with in-basket outcomes as continuous dependent variables, physician sex (female vs male) as the primary independent variable, and covariates that included academic rank, years in practice, mean patient age, mean number of problems per patient, and panel size. We included both academic rank and years in practice to capture distinct aspects of physician experience and considered collinearity diagnostics as acceptable. We derived adjusted marginal means and sex differences with 95% CIs from each model. We conducted an analysis in the full sample and in a subgroup restricted to nonprocedural specialties to assess robustness. We excluded physicians with incomplete covariate data since comparisons showed no meaningful differences between those with complete versus incomplete in-basket data. We defined statistical significance as 2-tailed *P*<.05 and performed analyses using JMP (SAS Institute).

## Results

As shown in Table 1, of 384 physicians with baseline data, sex was reported for 367 (n=146 female; n=221 male); 17 physicians with missing sex were excluded from sex-based analyses. Complete EHR in-basket data were available for 304 physicians (n=130 female; n=174 male). Of these, RVU information was available for 296 physicians (n=124 female; n=172 male), who were included in unadjusted RVU-normalized analyses. Adjusted analyses included 257 physicians (n=108 female; n=149 male) with complete covariate data.

Complete EHR in-basket burden data was available for 304 physicians (n=130 female; n=174 male). Physicians with complete in-basket data did not differ meaningfully from those with incomplete data with respect to physician sex, academic rank, years in practice, or patient panel characteristics. Table 2 presents adjusted marginal means with 95% CIs for female and male physicians, along with adjusted sex differences and associated *P* values. After adjustment for academic rank, years in practice, and panel characteristics, female physicians sustained higher RVU-normalized in-basket workloads than male physicians, suggesting that these differences are not driven solely by differences in the ratio of physicians in predominantly procedural subspecialties. In multivariable linear regression models, female physicians had higher adjusted mean daily total in-basket time, time spent completing messages, and EHR time outside 7 AM to 7 PM per RVU than male physicians. In contrast, adjusted differences in message counts were attenuated, with the largest and most consistent sex differences observed for staff messages per RVU. Significant differences in EHR in-basket burden metrics by sex persisted in analyses including all specialties and in analyses restricted to nonprocedural specialties (Table S1 in Multimedia Appendix 1).

**Table 1:** Internal medicine specialty physician, productivity, and patient panel characteristics.

Variables				Female physicians		Male physicians		*P* value
RVUs^a^, mean (SD)				534.92 (42.3)		666.1 (44.2)		.03
**Demographic characteristics, n (%)**								
	**Academic rank^b^**							<.001
		Total	146 (39.8)		221 (60.2)			
		Instructor	64 (43.8)		59 (26.7)			
		Assistant professor	53 (36.3)		66 (29.9)			
		Associate professor	22 (15.1)		51 (23.1)			
		Professor	7 (4.8)		45 (20.4)			
	**Years in practice^b^**							<.001
		Total	151 (41.1)		233 (63.5)			
		0-10 years	27 (17.9)		22 (9.4)			
		11-20 years	68 (45.3)		70 (30.0)			
		21-30 years	37 (24.5)		60 (25.8)			
		31 years or more	19 (12.6)		81 (34.8)			
**Panel characteristics, mean (SD)**								
	Patient age (years)			56.2 (9.9); n=113 patients		58.3 (8.8); n=153 patients		.07
	Problems per patient (n)			14.7 (4.7); n=113 patients		14.8 (4.1); n=153 patients		.84
	Patients per RVU (n)			0.37 (0.08); n=124 patients		0.21 (0.03); n=172 patients		.08
**EHR in-basket burden data, mean (SD)**								
	Daily time spent on in-basket per physician per RVU (minutes)			314 (32.6)		246 (25.1)		.02
	Daily time spent on completing messages per physician per RVU (minutes)			18,888.07 (1961.4)		14,808.09 (1507.6)		.02
	Daily time spent outside 7 AM-7 PM per physician per RVU (minutes)			113.2 (17.8)		101.2 (17.4)		.14
	Complete messages (n)			387.9 (40.3)		360.4 (33.2)		.17
	Staff messages (n)			49.1 (5.7)		34.3 (4.1)		.03
	Patient advice messages (n)			70.8 (9.13)		47.5 (5.9)		.007

^a^RVU: relative value unit.

^b^Percentantages in these columns use the value in the corresponding entry in the same column for Total under the same nested heading.

**Table 2:** Relative value unit (RVU)–normalized electronic health record in-basket burden metrics by physician sex. (Adjusted for physician demographics, patient characteristics, and panel size.)

In-basket efficiency metric	Unadjusted, normalized by RVU					Adjusted, normalized by RVU			
	Female (n=124), mean (SD; 95% CI)	Male (n=172, mean (SD; 95% CI)	Mean difference	*P* value	Female (n=108), mean (SD; 95% CI)		Male (n=149), mean (SD; 95% CI)	Mean difference	*P* value
Daily time spent on in-basket per RVU (min)	1.46 (1.83; 1.14-1.78)	0.80 (1.09; 0.64-0.96)	0.66	.001	1.27 (0.09; 1.25-1.29)		0.96 (0.08; 0.94-0.97)	0.31	<.001
Daily time spent on completing messages per RVU (min)	88.21(110.16; 68.82-107.6)	48.89 (65.98; 39.03-58.75)	39.3	<.001	76.40 (5.69; 75.4-77.4)		57.71 (4.65; 57.0-58.4)	18.7	.01
Daily time spent outside 7 AM-7 PM per RVU (min)	0.55 (0.79; 0.65-0.93)	0.29 (0.47; 0.22-0.36)	0.26	.002	0.44 (0.06; 0.43-0.45)		0.30 (0.05; 0.29-0.31)	0.14	.05
Messages per RVU (n)	1.36 (1.46; 1.10-1.62)	1.01 (0.93; 0.87-1.15)	0.35	.03	1.53 (0.12; 1.51-1.55)		1.41 (0.10; 1.39-1.43)	0.12	.44
Staff messages per RVU (n)	0.19 (0.21; 0.15-0.23)	0.10 (0.11; 0.08-0.12)	0.09	<.001	0.18 (0.01; 0.16-0.20)		0.12 (0.01; 0.1-0.14)	0.06	.002
Patient advice messages per RVU (n)	0.006 (0.006; 0.004-0.011)	0.004 (0.005; 0.003-0.011)	0.002	.03	0.005 (0.0009; 0.003-0.007)		0.006 (0.0008; 0.0048-0.0072)	0.001	.80

## Discussion

Female physicians spent more time on all in-basket activities and received a higher number of staff and patient messages per RVU. These findings persisted even after adjusting for physicians’ age, years in practice, and patient panel characteristics, and among procedural and nonprocedural specialties. Our results expand beyond primary care to internal medicine specialists, normalizing in-basket burden by RVU to emphasize that female physicians have a greater burden of in-basket work per unit of paid clinical care. These findings potentially reflect other studies on differential expectations for female physicians from patients and staff. However, our study demonstrates the pervasiveness of such sex-based differences across medical specialties, and notably, their impact on pay equity [3-5].

Limitations of our study could have influenced our findings. For instance, using data from a single academic medical center over a relatively short period could have reduced generalizability. Exclusion of physicians with incomplete data, who may have differed in unmeasured ways, could have biased the estimates. Use of RVUs could have incompletely captured clinical workloads. Finally, a lack of data on medical assistant support or clinical full-time equivalent hours was a confounder.

Our results have practical implications for physician well-being in ambulatory medical specialties. Compensation for in-basket activities would enable recognition of workload disparities. Use of artificial intelligence to automate message management could reduce inequities in uncompensated work. To address physician burnout, health systems must directly confront and mitigate sex disparities in EHR workload. Moreover, given physicians are currently not generally compensated for in-basket work, future studies should assess interventions to reduce sex differences in in-basket burden relative to compensated care delivered.

## Appendix

Multimedia Appendix 1 Relative value unit–normalized electronic health record in-basket burden metrics by physician sex and nonprocedural internal medicine specialties.

Multimedia Appendix 2 STROBE checklist.

## Abbreviations

EHR: electronic health record
RVU: relative value unit
