# Supplementary material for: Sex Differences in Medical Specialist Physicians’ Electronic Health Record In-Basket Workloads and the Implications for Compensation and Equity: Retrospective Cross-Sectional Study
Source: J Med Internet Res. 2026 Feb 18;28:e79172. doi: 10.2196/79172 (PMC12961384; doi:10.2196/79172)
Supplement: Multimedia Appendix 1 [file jmir_v28i1e79172_app1.docx]

**Table S1**. **RVU-normalized EHR inbox burden metrics by physician sex,**

**non-procedural internal medicine specialties**

|  | **Unadjusted Normalized by RVU** | | | **Adjusted Normalized by RVU** | | | |
| --- | --- | --- | --- | --- | --- | --- | --- |
| **In-Basket Efficiency Metric** | Female = 67  Mean(SD)  [95% CI] | Male = 62  Mean (SD)  [95% CI] | Mean  Difference  P-value | Female = 57  Mean (SD)  [95% CI] | Male = 54  Mean (SD)  [95% CI] | Mean  Difference  P-value |  |
| **Mean daily total time spent on in-basket per RVU (min/RVU)** | 1.90 (4.15)  [0.91-2.89] | 0.72 (0.62)  [0.55-0.89] | Diff=1.18  P=0.006 | 1.81 (1.07)  [1.53, 2.09] | 1.22 (1.11)  [0.93, 1.51] | Diff=0.59  P=0.008 |  |
| **Mean daily total time spent on completing messages per RVU (min/RVU)** | 113.66 (249.06)  [189.5-308.6] | 43.29 (37.25)  [34.3-52.5] | Diff=70.37  P=0.006 | 108.4 (65.7)  [91.31, 125.46] | 73.15 (65.8)  [55.56, 90.75] | Diff=35.23  P=0.009 |  |
| **Mean total daily time spent outside of 7am-7pm per RVU (min/RVU)** | 0.61 (1.20)  [0.32-0.89] | 0.22 (0.42)  [0.12-0.32] | Diff=0.39  P=0.066 | 0.61 (0.6)  [0.45, 0.77] | 0.35 (0.62)  [0.18, 0.51] | Diff=0.26  P=0.038 |  |
| **Total number of messages per RVU (msgs/RVU)** | 2.27 (6.86)  [0.63-3.9] | 0.98 (0.79)  [0.78-1.2] | Diff=1.29  P=0.109 | 2.03 (1.34)  [1.68, 2.38] | 1.74 (1.34)  [1.38, 2.10] | Diff=0.29  P=0.290 |  |
| **Total number of staff messages per RVU (msgs/RVU)** | 0.26 (0.68)  [0.09-0.42] | 0.10 (0.09)  [0.08-0.12] | Diff=0.16  P=0.005 | 0.22 (0.15)  [0.18, 0.27] | 0.18 (0.15)  [0.14, 0.23] | Diff=0.04  P=0.256 |  |
| **Total number of patient advice messages per RVU (msgs/RVU)** | 0.38 (0.68)  [0.22-0.54] | 0.15 (0.17)  [0.11-0.19] | Diff=0.23  P=0.023 | 0.40 (0.42)  [0.29, 0.51] | 0.20 (0.41)  [0.09, 0.31] | Diff=0.20  P=0.019 |  |
