# Supplementary material for: Sex Differences in Medical Specialist Physicians’ Electronic Health Record In-Basket Workloads and the Implications for Compensation and Equity: Retrospective Cross-Sectional Study
Source: J Med Internet Res. 2026 Feb 18;28:e79172. doi: 10.2196/79172 (PMC12961384; doi:10.2196/79172)
Supplement: Multimedia Appendix 2 [file jmir_v28i1e79172_app2.docx]

STROBE Statement—checklist of items that should be included in reports of observational studies

|  | Item No. | Recommendation | Page  No. | Relevant text from manuscript |
| --- | --- | --- | --- | --- |
| **Title and abstract** | 1 | (*a*) Indicate the study’s design with a commonly used term in the title or the abstract | 1 | As per title |
|  |  | (*b*) Provide in the abstract an informative and balanced summary of what was done and what was found | - | N/A (Minimal abstract for research letter) |
| Introduction | | | |  |
| Background/rationale | 2 | Explain the scientific background and rationale for the investigation being reported | 1 | Female physicians in primary care receive higher volumes of patient and staff messages in the electronic health record (EHR) in-basket [1,2]. However, little is known on whether sex differences extend into medical specialties and impact on workload disparities relative to compensated care. |
| Objectives | 3 | State specific objectives, including any prespecified hypotheses | 1 | In this cross-sectional study, we assessed sex differences in in-basket messages generated by outpatient workflows among internal medicine specialists as compared to relative value units (RVUs). We aimed to clarify whether female medical specialty physicians experience disproportionately greater uncompensated electronic work for each unit of paid clinical care. |
| Methods | | | |  |
| Study design | 4 | Present key elements of study design early in the paper | 1 |  |
| Setting | 5 | Describe the setting, locations, and relevant dates, including periods of recruitment, exposure, follow-up, and data collection | 1 | Under “Methods” including “Study Procedures” : We conducted the study at a large, academic medical center. All attending physicians actively practiced in outpatient specialty practices with measurable in-basket activity during the study period… We collected monthly, ambulatory in-basket physician data and patient characteristics for each attending physician’s patient panel from Epic for January through March 2021 and normalized monthly in-basket burden measures by monthly RVUs. Patients are generally assigned to physicians based on availability and patient preference. We obtained physician sex, academic rank, and practice years from the public institutional directory, which reports sex as male or female; no physicians were listed as nonbinary. |
| Participants | 6 | (*a*) *Cohort study*—Give the eligibility criteria, and the sources and methods of selection of participants. Describe methods of follow-up  *Case-control study*—Give the eligibility criteria, and the sources and methods of case ascertainment and control selection. Give the rationale for the choice of cases and controls  *Cross-sectional study*—Give the eligibility criteria, and the sources and methods of selection of participants | 1 | Under “Methods” and “Study Procedures” We collected monthly, ambulatory in-basket physician data and patient characteristics for each attending physician’s patient panel from Epic for January through March 2021 and normalized monthly in-basket burden measures by monthly RVUs. Patients are generally assigned to physicians based on availability and patient preference. We obtained physician sex, academic rank, and practice years from the public institutional directory, which reports sex as male or female; no physicians were listed as nonbinary. |
|  |  | (*b*) *Cohort study*—For matched studies, give matching criteria and number of exposed and unexposed  *Case-control study*—For matched studies, give matching criteria and the number of controls per case | N/A | N/A |
| Variables | 7 | Clearly define all outcomes, exposures, predictors, potential confounders, and effect modifiers. Give diagnostic criteria, if applicable | 2 | Statistical analyses: We compared continuous variables using Wilcoxon rank sum tests, and categorical variables using chi-square tests or Fisher exact tests when expected cell counts were small. To assess sex differences in in-basket burden, we fit multivariable linear regression models with in-basket outcomes as continuous dependent variables, physician sex (female vs male) as the primary independent variable, and covariates that included academic rank, years in practice, mean patient age, mean number of problems per patient, and panel size. We included both academic rank and years in practice to capture distinct aspects of physician experience and considered collinearity diagnostics as acceptable. We derived adjusted marginal means and sex differences with 95% CIs from each model. We conducted an analysis in the full sample and in a subgroup restricted to nonprocedural specialties to assess robustness. We excluded physicians with incomplete covariate data since comparisons showed no meaningful differences between those with complete versus incomplete in-basket data. We defined statistical significance as 2-tailed *P*<.05 and performed analyses using JMP (SAS Institute). |
| Data sources/ measurement | 8* | For each variable of interest, give sources of data and details of methods of assessment (measurement). Describe comparability of assessment methods if there is more than one group | 1 | We collected monthly, ambulatory in-basket physician data and patient characteristics for each attending physician’s patient panel from Epic for January through March 2021 and normalized monthly in-basket burden measures by monthly RVUs. Patients are generally assigned to physicians based on availability and patient preference. We obtained physician sex, academic rank, and practice years from the public institutional directory, which reports sex as male or female; no physicians were listed as nonbinary. We categorized specialties as those including a substantial procedural component (cardiology, gastroenterology, and pulmonology) versus those predominantly not procedural (genetics, geriatrics, hematology, immunology, infectious disease, nephrology, palliative care, rheumatology, and sleep medicine). |
| Bias | 9 | Describe any efforts to address potential sources of bias | 4-5 | Table 2: Adjusted for physician demographics, patient characteristics, and panel size |
| Study size | 10 | Explain how the study size was arrived at | 1 | We collected monthly, ambulatory in-basket physician data and patient characteristics for each attending physician’s patient panel from Epic for January through March 2021 and normalized monthly in-basket burden measures by monthly RVUs. Patients are generally assigned to physicians based on availability and patient preference. We obtained physician sex, academic rank, and practice years from the public institutional directory, which reports sex as male or female; no physicians were listed as nonbinary. |

Continued on next page

| Quantitative variables | 11 | Explain how quantitative variables were handled in the analyses. If applicable, describe which groupings were chosen and why | 2 | Study analyses: We compared continuous variables using Wilcoxon rank sum tests, and categorical variables using chi-square tests or Fisher exact tests when expected cell counts were small. To assess sex differences in in-basket burden, we fit multivariable linear regression models with in-basket outcomes as continuous dependent variables, physician sex (female vs male) as the primary independent variable, and covariates that included academic rank, years in practice, mean patient age, mean number of problems per patient, and panel size. We included both academic rank and years in practice to capture distinct aspects of physician experience and considered collinearity diagnostics as acceptable |
| --- | --- | --- | --- | --- |
| Statistical methods | 12 | (*a*) Describe all statistical methods, including those used to control for confounding | 2 | We compared continuous variables using Wilcoxon rank sum tests, and categorical variables using chi-square tests or Fisher exact tests when expected cell counts were small. To assess sex differences in in-basket burden, we fit multivariable linear regression models with in-basket outcomes as continuous dependent variables, physician sex (female vs male) as the primary independent variable, and covariates that included academic rank, years in practice, mean patient age, mean number of problems per patient, and panel size. We included both academic rank and years in practice to capture distinct aspects of physician experience and considered collinearity diagnostics as acceptable. We derived adjusted marginal means and sex differences with 95% CIs from each model. We conducted an analysis in the full sample and in a subgroup restricted to nonprocedural specialties to assess robustness. We excluded physicians with incomplete covariate data since comparisons showed no meaningful differences between those with complete versus incomplete in-basket data. We defined statistical significance as 2-tailed *P*<.05 and performed analyses using JMP (SAS Institute). |
|  |  | (*b*) Describe any methods used to examine subgroups and interactions | 2 | Statistical analyses: We compared continuous variables using Wilcoxon rank sum tests, and categorical variables using chi-square tests or Fisher exact tests when expected cell counts were small. To assess sex differences in in-basket burden, we fit multivariable linear regression models with in-basket outcomes as continuous dependent variables, physician sex (female vs male) as the primary independent variable, and covariates that included academic rank, years in practice, mean patient age, mean number of problems per patient, and panel size. We included both academic rank and years in practice to capture distinct aspects of physician experience and considered collinearity diagnostics as acceptable. We derived adjusted marginal means and sex differences with 95% CIs from each model. We conducted an analysis in the full sample and in a subgroup restricted to nonprocedural specialties to assess robustness. We excluded physicians with incomplete covariate data since comparisons showed no meaningful differences between those with complete versus incomplete in-basket data. We defined statistical significance as 2-tailed *P*<.05 and performed analyses using JMP (SAS Institute). |
|  |  | (*c*) Explain how missing data were addressed | 2 | We excluded physicians with incomplete covariate data since comparisons showed no meaningful differences between those with complete versus incomplete in-basket data. |
|  |  | (*d*) *Cohort study*—If applicable, explain how loss to follow-up was addressed  *Case-control study*—If applicable, explain how matching of cases and controls was addressed  *Cross-sectional study*—If applicable, describe analytical methods taking account of sampling strategy | N/A | N/A |
|  |  | (*e*) Describe any sensitivity analyses | 2 | We conducted an analysis in the full sample and in a subgroup restricted to nonprocedural specialties to assess robustness. We excluded physicians with incomplete covariate data since comparisons showed no meaningful differences between those with complete versus incomplete in-basket data. |
| Results | | | | |
| Participants | 13* | (a) Report numbers of individuals at each stage of study—eg numbers potentially eligible, examined for eligibility, confirmed eligible, included in the study, completing follow-up, and analysed | 2 | Results section: Of 384 physicians with baseline data, sex was reported for 367 (146 female, 221 male); 17 physicians with missing sex were excluded from sex-based analyses. Complete EHR in-basket data were available for 304 physicians (130 female, 174 male). Of these, RVU information was available for 296 physicians (124 female, 172 male), who were included in unadjusted RVU-normalized analyses. Adjusted analyses included 257 physicians (108 female, 149 male) with complete covariate data. |
|  |  | (b) Give reasons for non-participation at each stage | 2 | Results section: Of 384 physicians with baseline data, sex was reported for 367 (146 female, 221 male); 17 physicians with missing sex were excluded from sex-based analyses. Complete EHR in-basket data were available for 304 physicians (130 female, 174 male). Of these, RVU information was available for 296 physicians (124 female, 172 male), who were included in unadjusted RVU-normalized analyses. Adjusted analyses included 257 physicians (108 female, 149 male) with complete covariate data. |
|  |  | (c) Consider use of a flow diagram | N/A | N/A |
| Descriptive data | 14* | (a) Give characteristics of study participants (eg demographic, clinical, social) and information on exposures and potential confounders | 2-3 | Table 1 |
|  |  | (b) Indicate number of participants with missing data for each variable of interest | 2 | Of 384 physicians with baseline data, sex was reported for 367 (146 female, 221 male); 17 physicians with missing sex were excluded from sex-based analyses. Complete EHR in-basket data were available for 304 physicians (130 female, 174 male). Of these, RVU information was available for 296 physicians (124 female, 172 male), who were included in unadjusted RVU-normalized analyses. Adjusted analyses included 257 physicians (108 female, 149 male) with complete covariate data. |
|  |  | (c) *Cohort study*—Summarise follow-up time (eg, average and total amount) | N/A | N/A |
| Outcome data | 15* | *Cohort study*—Report numbers of outcome events or summary measures over time | N/A | N/A |
|  |  | *Case-control study—*Report numbers in each exposure category, or summary measures of exposure | N/A | N/A |
|  |  | *Cross-sectional study—*Report numbers of outcome events or summary measures | 4-5 | Table 2 |
| Main results | 16 | (*a*) Give unadjusted estimates and, if applicable, confounder-adjusted estimates and their precision (eg, 95% confidence interval). Make clear which confounders were adjusted for and why they were included | 4-5 | Table 2 |
|  |  | (*b*) Report category boundaries when continuous variables were categorized | 2-3 | Table 1 |
|  |  | (*c*) If relevant, consider translating estimates of relative risk into absolute risk for a meaningful time period | N/A | N/A |

Continued on next page

| Other analyses | 17 | Report other analyses done—eg analyses of subgroups and interactions, and sensitivity analyses | 4-5 | Table 2 |
| --- | --- | --- | --- | --- |
| Discussion | | | | |
| Key results | 18 | Summarise key results with reference to study objectives | 5 | Discussion Section: Female physicians spent more time on all in-basket activities and received a higher number of staff and patient messages per RVU. These findings persisted even after adjusting for physicians’ age, years in practice, and patient panel characteristics, and among procedural and nonprocedural specialties. Our results expand beyond primary care to internal medicine specialists, normalizing in-basket burden by RVU to emphasize that female physicians have a greater burden of in-basket work per unit of paid clinical care. |
| Limitations | 19 | Discuss limitations of the study, taking into account sources of potential bias or imprecision. Discuss both direction and magnitude of any potential bias | 5 | Discussion Section: Limitations of our study could have influenced our findings. For instance, using data from a single academic medical center over a relatively short period could have reduced generalizability. Exclusion of physicians with incomplete data, who may have differed in unmeasured ways, could have biased the estimates. Use of RVUs could have incompletely captured clinical workloads. Finally, a lack of data on medical assistant support or clinical full-time equivalent hours was a confounder. |
| Interpretation | 20 | Give a cautious overall interpretation of results considering objectives, limitations, multiplicity of analyses, results from similar studies, and other relevant evidence | 5 | Discussion Section: These findings potentially reflect other studies on differential expectations for female physicians from patients and staff. However, our study demonstrates the pervasiveness of such sex-based differences across medical specialties, and notably, their impact on pay equity [3-5]. |
| Generalisability | 21 | Discuss the generalisability (external validity) of the study results | 5 | Discussion Section: Our results expand beyond primary care to internal medicine specialists, normalizing in-basket burden by RVU to emphasize that female physicians have a greater burden of in-basket work per unit of paid clinical care. These findings potentially reflect other studies on differential expectations for female physicians from patients and staff. However, our study demonstrates the pervasiveness of such sex-based differences across medical specialties, and notably, their impact on pay equity [3-5]. |
| Other information | |  | | |
| Funding | 22 | Give the source of funding and the role of the funders for the present study and, if applicable, for the original study on which the present article is based | 6 | Acknowledgements Section |

*Give information separately for cases and controls in case-control studies and, if applicable, for exposed and unexposed groups in cohort and cross-sectional studies.

**Note:** An Explanation and Elaboration article discusses each checklist item and gives methodological background and published examples of transparent reporting. The STROBE checklist is best used in conjunction with this article (freely available on the Web sites of PLoS Medicine at http://www.plosmedicine.org/, Annals of Internal Medicine at http://www.annals.org/, and Epidemiology at http://www.epidem.com/). Information on the STROBE Initiative is available at www.strobe-statement.org.
